# The Urine in Health and Disease, and Urinary Analysis

**Published:** 1895-06

**Authors:** 


*The Urine in Health and Disease, and Urinary Analysis.* By D. CAMPBELL BLACK, M.D. Pp. xiii., 246. London: BAILLIÈRE, TINDALL AND COX. 1895.

This work begins with a concise description of the structure of the kidney and its physiology. The first part contains a full account of the normal elements of the urine, very scientifically treated and satisfactory in every way. The next part deals equally fully with the abnormal substances found in urine. The tests for albumen are carefully described, and ample information given as to the practical value of each test. There is a useful table of the relative delicacy of the tests obtained by determining the smallest amount of albumen each will respectively indicate: we would suggest that in future editions a scheme should be appended directing the student as to the sequence in which the tests should be employed in order to distinguish the particular variety of proteid present. Seeing the value of phenyl-hydrazine reaction for sugar and that it is a new test, it would have been helpful to those unacquainted with the reaction if there had been a picture of the phenyl-glucosazine crystals. Further, a little more advice as to the best tests for sugar to employ in daily practice is desirable. But these are very minor faults. While if it does not contain anything original, we can recommend the book as an excellent summary of the subject with which it deals.

